# The influence of recent COVID-19 infection on patients undergoing thyroid surgery: Clinical outcomes and patient perception observations

**DOI:** 10.1097/MD.0000000000046400

**Published:** 2026-05-12

**Authors:** Mengjia Fei, Yuan Shi, Kai Qian, Kai Guo, Zhuoying Wang

**Affiliations:** a Department of Head and Neck Surgery, Renji Hospital, School of Medicine, Shanghai Jiao Tong University, Shanghai, China.

**Keywords:** COVID-19, delay of surgery, patient perceptions, SARS-CoV-2 IgG, thyroid surgery

## Abstract

Our research observed the condition and potential surgical risks of patients who have previously contracted coronavirus disease 2019 (COVID-19) and subsequently underwent thyroid surgery. A research cohort of 140 patients undergoing thyroid surgery within our institution between October 2022 and March 2023. Patients were categorized into 4 distinct groups, factoring in varying stages post their COVID-19 infection. The study documented various pre- and postoperative clinical data, as well as the patients’ subjective experiences. There existed no statistically significant distinction in the incidence of postoperative adverse reactions (*P* = .81) or the pharyngalgia score (*P* = .57) among the different patient groups. The statistical analysis revealed the severe acute respiratory syndrome-CoV-2 Immunoglobulin G (IgG) levels emerged as a critical positive correlation factor, signifying an escalated probability of postoperative adverse events (*r* = 0.31, *P* = .02). However, the levels of IgG are not affected by the extent of vaccination, and it do not exert a significant impact on postoperative pharyngalgia, pulmonary focal exudate, or tumor progression. Furthermore, the probability of postoperative adverse reactions heightened when patients still exhibited incomplete absorption of lung infection lesions on preoperative lung CT (*r* = 0.11, *P* = .04). With a thorough preoperative assessment, early thyroid surgery following COVID-19 infection is feasible. It is recommended to incorporate IgG testing as an indicator for assessing adverse surgical events.

## 1. Introduction

The widespread outbreak of coronavirus disease 2019 (COVID-19) has exerted significant pressure on the global healthcare infrastructure. Responding to this medical exigency, medical associations in the United States, Europe, and China have issued and continually updated a series of guidelines pertaining to the categorization and management of tumor surgeries.^[1–5]^ Due to the comparatively favorable prognosis of thyroid cancer vis-à-vis other solid organ cancers, these guidelines advocate for the postponement or cancelation of planned elective surgeries, including those for thyroid and select head and neck tumors. Thyroid cancer stands as one of the most prevalent endocrine malignancies globally.^[6]^ Various reports from around the world during different phases of the COVID-19 pandemic have indicated a reduction in thyroid tumor surgeries ranging from 2.3% to 64.8%.^[4,7–10]^

It is evident that patients grappling with thyroid cancer, particularly those who have also battled a COVID-19 infection, face markedly elevated risks of complications or even mortality upon undergoing surgical procedures compared to their non-COVID-19 afflicted counterparts. Amidst the ongoing Omicron variant pandemic, we confront 2 critical inquiries concerning patients who have surpassed the infectious stage during their recovery period. What are the perilous implications for patients necessitating thyroid tumor surgery? What is the optimal interval between convalescence from COVID-19 and the surgical intervention? These inquiries necessitate prompt elucidation. Currently, deliberations are ongoing regarding the timing of surgical intervention for patients previously infected with the virus.^[2,4,7,11,12]^ In accordance with the stratification of symptoms and their severity, it is advisable to defer surgical treatment until 4 weeks after the diagnosis of severe acute respiratory syndrome (SARS)-CoV-2 infection for asymptomatic or mildly symptomatic cases. In most other instances, it is prudent for patients to contemplate surgical procedures at least 7 weeks post their COVID-19 diagnosis. An international multicenter study revealed a persistent heightened risk of perioperative mortality within the initial 6 weeks following COVID-19 infection.^[9]^ Additionally, a comprehensive surgical study disclosed that the incidence of postoperative complications such as pneumonia, respiratory failure, pulmonary embolism, sepsis, and others was at its lowest for patients in the surgical group after 8 weeks of infection.^[2,7,9,13]^ Naturally, it’s important to note that many of the aforementioned studies are predicated on data from the early stages of the novel coronavirus epidemic, and they do not differentiate whether the risks for vaccinated patients vary. Presently, the prevailing strain of the pandemic is the Omicron variant, characterized by a slightly abbreviated incubation period and milder pulmonary impairment.^[12,14,15]^ However, robust statistical data evaluating post-thyroid tumor surgery outcomes in patients recovering from recent Omicron variant infections remains conspicuously absent.

The geographical location of our research center recently witnessed a brief yet extensive propagation of the Omicron mutant in December 2022. This study endeavors to compile clinical data from patients who underwent thyroid tumor surgery at the center following this brief but intensive localized Omicron transmission. This effort, combined with the subjective perspectives of post-surgery patients and a comprehensive summary of COVID-19 symptoms experienced during infection, seeks to meticulously observe and analyze the traits of thyroid tumor patients undergoing surgery at varying intervals post-infection. The objective is to ascertain the optimal conditions for infected patients to undergo surgery successfully, delineating an appropriate preoperative waiting period.

## 2. Materials and methods

### 2.1. Patient cohort and data acquisition

A cohort of 105 convalescent COVID-19 patients who underwent surgical procedures for thyroid cancer at our institution between January and March 2023 were enrolled in this investigation. The COVID-19 diagnosis was confirmed through viral nucleic acid testing and SARS-CoV-2 antigen testing during a period of intense and widespread local infection. The patients were categorized into distinct groups: group 1, termed the peri-COVID-19 surgery group, comprised individuals who underwent surgeries within 2 to 4 weeks after the COVID-19 diagnosis; group 2, identified as the early post-COVID-19 surgery group, encompassed patients who underwent surgery within 4 to 8 weeks post the COVID-19 diagnosis; group 3, designated the late post-COVID-19 surgery group, included individuals whose surgery took place 8 weeks or more after the COVID-19 diagnosis. The fourth group comprised another 35 patients from our center who underwent surgery between October and November 2022: Their inclusion in the control group was confirmed through a review of their medical history, validating no exposure to or infection with COVID-19 for a period exceeding 6 months, including their cohabitants. No surgeries were performed within the first 2 weeks (0–14 days) post COVID-19 diagnosis, and no emergency cases were included. Comprehensive patient information was extracted from the electronic medical record system.

The study was conducted in accordance with the Declaration of Helsinki and approved by the Ethics Committee of Renji Hospital, School of Medicine, Shanghai Jiao Tong University (approval AF-2022-198). Parents provided a written informed consent.

### 2.2. Preoperative screening and assessment

All prospective surgery patients underwent a meticulous assessment for exposure to SARS-CoV-2 and associated symptoms within the initial fortnight. This encompassed an evaluation of symptoms like fever, cough, dyspnea, myalgia, sore throat, and alterations in taste or smell. Given the prevailing widespread community infections during the study timeframe, patients were subject to an extensive evaluation at the outpatient department 1 to 2 days prior to admission. This evaluation necessitated lung CT scans, lung function tests, and other assessments to ascertain the absence of evident pulmonary inflammation before admission. Additionally, patients were subjected to polymerase chain reaction (PCR) testing for SARS-CoV-2 specific RNA 24 hours before the scheduled surgery to confirm their SARS-CoV-2 infection status. A negative result was a prerequisite for admission, and the surgical scheduling was contingent on antigen testing on the day of the surgery.

### 2.3. Data collection and antibody quantification

The study gathered comprehensive clinical information regarding tumors and treatment modalities of the patients. To augment data collection, a tailored questionnaire was formulated, eliciting patients’ responses concerning non-respiratory symptoms post-surgery, encompassing parameters such as sore throat, cough, difficulty in breathing, duration, and weakness (Supplementary Files, Supplemental Digital Content, https://links.lww.com/MD/Q842). Additionally, the type and dosage of the administered COVID-19 vaccine were diligently recorded.

Serum supernatant derived from patients was procured and utilized for antibody detection. Serial measurements of SARS-CoV-2 anti-nucleocapsid and anti-spike Immunoglobulin M (IgM)/Immunoglobulin G (IgG) were conducted utilizing iFlash-SARS-CoV-2 (YHLO Biotechnology Co., Ltd, Shenzhen, China), a chemiluminescence immunoassay based on paramagnetic particles. According to the manufacturer’s specifications, the IgM and IgG cutoff value was set at 10.0 kAU/L. Lung Ct, IgG, and IgM levels were taken into account to establish any potential correlations between anti-SARS-CoV-2 antibodies and postoperative syndrome.

### 2.4. Statistical analysis

Patients were categorized into 4 groups based on time intervals and their COVID-19 history during the interview. Various statistical measures, including absolute values, percentages, mean, and median (standard deviation or interquartile range), were computed. Categorical variables were compared utilizing the chi-squared test or Fisher’s exact test, while continuous variables were assessed using the Student *t* test or Mann–Whitney *U* test for 2 groups, and the 1-way ANOVA test for comparisons involving more than 2 groups. Bonferroni correction was used for post hoc multiple comparisons. Correlations between factors associated with COVID-19 and factors related to thyroid surgery were analyzed using the Spearman correlation coefficient. Statistical significance was defined at *P* values < .05. All statistical analyses were performed using SPSS Statistics 22 (IBM® SPSS® Statistics version 22.0, Armonk).

## 3. Results

### 3.1. Baseline patient characteristics

In total, throughout the study duration, 105 convalescent COVID-19 patients who underwent thyroid tumor surgery at our facility were included in the study. Likewise, another 35 patients devoid of any history of associated infections, who had undergone surgery in our hospital, met the specified inclusion criteria for participation in this study. An overview of the fundamental demographic and clinical characteristics of the patients is presented in Table 1. In summary, following the statistical analysis of basic patient information across the 4 distinct patient groups, it was ascertained that the average age, gender distribution, and BMI of patients at various stages post-COVID infection and subsequent surgery did not exhibit any statistically significant variations.

**Table 1:** Baseline demographic and clinical characteristics of the 4 patient groups.

	Peri-Covid-19 surgery 2–4 wk after Covid-19	Early post-Covid-19 surgery 4–8 wk after Covid-19	Late post-Covid-19 surgery at least 8 wk after Covid-19	Pre-Covid-19 surgery before December 1, 2022	*P* value
Number of patients	29	37	39	35	
Age ± SD	49.0 ± 10.4	50.1 ± 11.2	48.9 ± 10.9	50.9 ± 10.7	.77
Sex					.81
Male	7	9	8	8	
Female	22	26	25	27	
BMI ± SD (kg/m^2^)	23.2 ± 3.3	23.5 ± 3.4	23.3 ± 3.1	23.2 ± 2.9	.81
Average length of stay (d)	2.4	2.3	2.4	2.3	
Tumour stage					.53
I	19	22	18	19	
II	7	8	8	7	
III	2	4	5	6	
IV	1	1	2	3	
SARS-CoV-2 IgM (AU/mL ± SD)	1.2 ± 0.7*^,^†	0.3 ± 0.2	0.1 ± 0	NA	.02
SARS-CoV-2 IgG (AU/mL ± SD)	126.2 ± 78.9	91.2 ± 61.5	89.9 ± 55.3	NA	.34
Symptom grading on set					.02
Asymptomatic	27†	29	19	NA	
Slight	2†	5	11	NA	
Moderate and above	0†	1	3	NA	
Postoperative pharyngalgia (day 1)					.57
0	9	7	7	8	
1	11	13	15	16	
2	17	14	10	9	
3	0	1	3	2	
Postoperative adverse events, n (%)	3	2	3	3	.81

BMI = body mass index, COVID-19 = coronavirus disease 2019, SD = standard deviation.

* *P* < .05 vs early post-COVID-19 surgery group.

† *P* < .05 vs late post-COVID-19 surgery group.

In terms of medical treatment, there was no difference in the mean hospitalization duration or the distribution of tumor stages among patients undergoing surgery at various peri- and post-COVID-19 stages within our institution’s dataset. Moreover, <pharyngalgia>, denoting the prevalent discomfort following thyroid surgery, was employed as a standardizing parameter. Patients self-assessed the level of pharyngeal pain based on their subjective experiences. The outcomes revealed a lack of significant disparity in the postoperative ordeal for patients, irrespective of their SARS-CoV-2 infection status or the time elapsed since infection (*P* = .57). Additionally, the incidence of postoperative adverse events was similar between the 4 groups (*P* = .81).

### 3.2. The discrepancy in SARS-CoV-2 IgG antibody levels holds clinical significance

Every patient underwent serological assessments for anti-SARS-CoV-2 IgG and IgM antibodies on the eve of their respective surgeries. The IgM antibody levels of the majority of patients fell below the critical threshold (10 AU/mL), and the average level of SARS-CoV-2 IgM antibodies demonstrated a notable reduction over time (*P* = .02). Conversely, the average IgG antibody level in the majority of patients surpassed the critical threshold, and these IgG levels exhibited no significant alterations in relation to the duration since infection (*P* = .34). Furthermore, there was no discernible difference in IgG levels between male and female convalescent patients (Table 1). All 105 patients were segregated into 2 groups based on their IgG levels. Further classification was based on whether their IgG levels exceeded or were below the median Ig value of 79.3 AU/m (Table 2). Upon comparing full/partial vaccination (*P* = .43), postoperative pharyngeal discomfort (*P* = .82), the proportion of pulmonary focal exudate (by CT scan of lung; *P* = .79), and the degree of tumor malignancy (*P* = .65) between the 2 groups, it was evident that IgG levels did not exert a discernible influence on the outcomes. However, the likelihood of experiencing adverse events in patients with high IgG levels was nearly sevenfold greater compared to those in the low IgG group.

**Table 2:** Analysis of pertinent influencing factors based on varying IgG levels.

	High IgG group	Low IgG group	*P* value
Vaccine inoculation status			.43
Never, n (%)	4 (7.5%)	7 (13.5%)	
Partially vaccination, n (%)	3 (5.6%)	2 (3.8%)	
Whole course vaccination, n (%)	36 (67.9%)	43 (82.7%)	
Postoperative adverse events, n	7 (13.2%)	1 (1.9%)	.02
Healing of incision problems	3	1	
Delayed discharge	2	0	
Others	1	0	
Pharyngalgia scores ± SD	2.4 ± 0.47	2.1 ± 0.35	.82
Lung CT focal exudative, n (%)	19 (35.8%)	15 (28.8%)	.69
Tumour stage III and IV, n (%)	7 (13.5%)	10 (19.2%)	.65
Associated with Hashimoto thyroiditis	10 (18.9%)	11 (21.2%)	.89

CT = computed tomography, SD = standard deviation.

**Figure 1.**
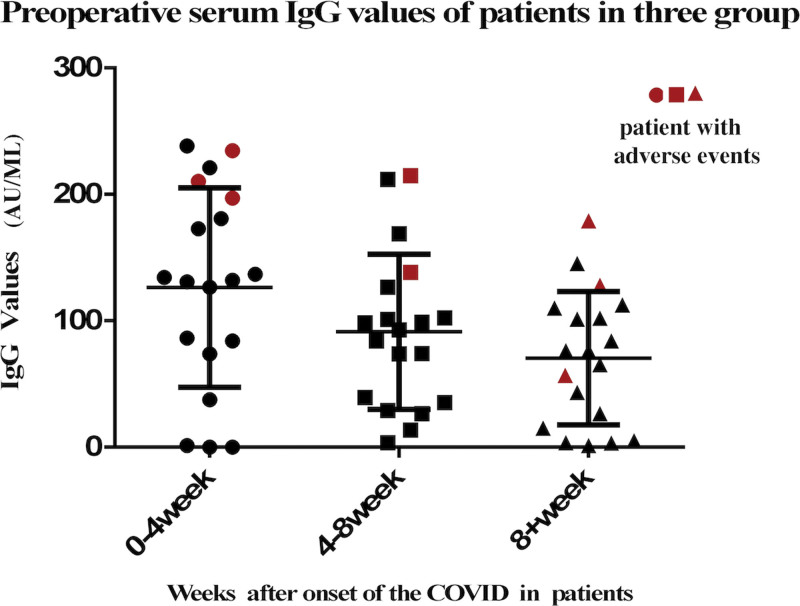
Anti-SARS-CoV-2 IgG levels in patients experiencing adverse events in 3 groups. COVID = coronavirus disease.

Display the IgG values for 3 groups of patients with a history of COVID-19 infection and mark those who experienced postoperative adverse events. Our observations reveal that despite variations in the interval between surgery and COVID-19 infection across the groups, individuals manifesting adverse events tended to exhibit comparatively elevated IgG levels within their respective groups (Fig. 1).

### 3.3. The influence of various factors on thyroid surgery

**Figure 2.**
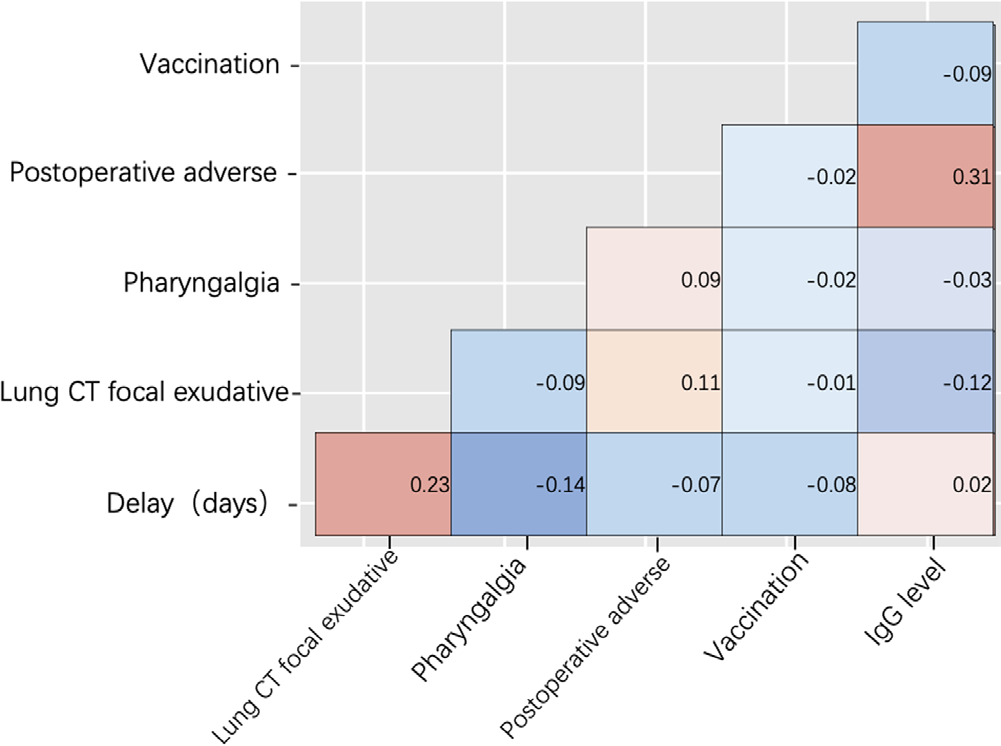
Correlations between variables of interest. The values represent the Pearson correlation coefficient (*r*), showing the strength of correlation (Corr). Corr = correlation, CT = computed tomography.

For the patient cohort with a history of COVID-19 infection, we examined the interrelation of several pertinent variables, as illustrated in Figure 2 (vacation status, presence of postoperative adverse events, severity of pharyngalgia, lung CT focal exudate, delay of admission, IgG levels). The primary determinant affecting the delay in patients’ hospitalization (*r* = 0.23, *P* < .01) was identified as the presence of local exudative manifestations in lung CT screenings. Pharyngalgia emerged as a representative discomfort following thyroid surgery, exhibiting a slight negative correlation with the delay in patients’ admission time (*r* = −0.14), although statistical differences were not evident (*P* = .27). Other factors’ influence on postoperative pharyngalgia was considered negligible. The overall incidence of postoperative adverse events in thyroid tumor cases was low, and its occurrence was correlated with the patient’s serum IgG levels in this study (*r* = 0.31, *P* < .01). Additionally, a weak positive correlation was observed between the incidence of postoperative adverse events and the presence of local exudative manifestations in lung CT screenings (*r* = 0.11, *P* = .03). The administration of the COVID-19 vaccine appears to offer some modest assistance in postoperative recovery, although its effects are relatively limited.

## 4. Discussion

Despite the absence of established protocols for managing patients necessitating thyroid surgery during the COVID-19 pandemic, a prudent approach was adopted. The urgency of surgical intervention was assessed based on a thorough analysis of tumor risk and its potential to progress to a life-threatening stage.^[1]^ A recent worldwide research suggest that the reduction in surgical activities for thyroid nodules of uncertain nature during the pandemic may lead to an increase in aggressive thyroid tumors.^[16]^ Another recent prospective cohort study (MAeSTro) from South Korea showed the delayed surgery group had a higher rate of lymph node dissection. The pathological data of MAeSTro revealed that the delayed surgery group had a larger tumor diameter, a higher lymph node metastasis rate and a more common multifocal tumor.^[17]^ Publication of these research results emphasizes the importance of exploring strategies that allow for early and safe surgery for patients after COVID-19 infection.

An integral aspect of our research involves the comparative evaluation of various parameters among patients undergoing surgery at different time intervals after contracting COVID-19. Our results suggests that, even in the early stages of infection, specifically during the initial 2 to 4 week peri-COVID-19 Surgery period, the incidence of postoperative complications does not exhibit a significant increase when compared to patients who undergo surgery after an 8-week waiting period. Furthermore, early surgical intervention does not lead to a significant prolongation of hospitalization duration. While patients who undergo surgery earlier tend to experience a higher frequency of postoperative sore throat, statistical differences remain nonsignificant. Noteworthy statistical disparities in tumor prognosis and infection-associated prognoses were not observed across patients at varying postinfection stages. This can be attributed to the meticulous approach in patient admission, overseen by outpatient physicians. The admission process involved a comprehensive evaluation, including lung CT scans and consultations, to ascertain the patients’ suitability for surgery. Consequently, a proportion of patients potentially prone to surgical and pulmonary complications were prudently deferred from inclusion in our investigation. Given the exceedingly rare incidence of perioperative mortality in thyroid surgery,^[18]^ our investigation predominantly delves into the postoperative encounter of patients and the adept management of complications. Our research ultimately aims to ascertain the earliest and safest timing for thyroid surgery in patients who have undergone COVID-19 infection, considering various physiological conditions. The thyroid gland’s anatomical proximity to the upper respiratory tract renders the direct stimulation from surgery notably pronounced in individuals with compromised lung and lower respiratory tract conditions. Subjective sensations like pharyngalgia was chosen as a crucial evaluation criterion, given that pharyngalgia ins a prevalent and foremost discomfort experienced post SARS-CoV-2 infection and thyroid surgery. The patient questionnaire indicated a gradual reduction in postoperative pharyngeal pain intensity as the waiting period for surgery extended. Nevertheless, we did not identify a significant correlation between individual postoperative pharyngalgia and pain experienced during SARS-CoV-2 infection. This discrepancy might stem from the predominantly mild sore throat response exhibited by the patients included in the study during their SARS-CoV-2 infection. We performed a correlation analysis encompassing multiple evaluation criteria derived from patients’ pharyngalgia surveys and postoperative complications. The statistical outcomes emphasize that preoperative IgG levels hold promise as an independent predictive indicator for the postoperative recovery of thyroid cancer surgeries.

Amongst patients experiencing postoperative adverse events, a notable observation was the markedly elevated anti-SARS-CoV-2 IgG antibody titers, surpassing the average levels. This finding appears to contradict our initial assumption of its protective effect. However, aligning with our conclusion, certain research outcomes substantiate this: a 6-month follow-up study on previously infected patients revealed that a sustained high titer of serological response to SARS-CoV-2 could independently pose a risk for the disease. This was accompanied by a prolonged persistence of discomfort symptoms and a heightened rate of ICU admissions postinfection.^[19]^ It’s pertinent to note that most of the aforementioned studies primarily focused on the early strains, known for their elevated pathogenicity and mortality rates. Nonetheless, we advocate exercising caution for patients with a history of COVID infection. Alongside lung CT scans and clinical symptom evaluations, particular attention should be given to those still exhibiting heightened IgG antibody levels at least 4 to 8 weeks postinfection. We also found that the vaccination status of patients does not exert a significant influence on their IgG levels. Furthermore, there exists no notable correlation between vaccination status and postoperative discomfort symptoms. Recent literature aligns with our findings, suggesting an absence of a substantial link between vaccination and various discomfort symptoms postinfection.^[20,21]^ Upon segmentation based on serum IgM and IgG antibody levels, no discernible difference was observed between male and female patients during the recovery period.^[22]^ However, in contrast, a substantial cohort study analyzed patients who underwent surgery within the early phase (first 4 weeks) post-COVID infection. The research revealed that for patients who did not receive the complete SARS-CoV-2 vaccination, the incidence of postoperative complications was higher compared to vaccinated patients. Intriguingly, this effect was not observed during surgeries conducted under local anesthesia.^[13]^

Controversy surrounds the optimal timing for neck surgery following a COVID-19 infection. Central to this deliberation is the proximity of the thyroid gland to critical structures like the trachea, esophagus, and major blood vessels in the neck, which could exacerbate postoperative discomfort symptoms – such as sore throat, respiratory challenges, and swallowing difficulties. In our study, we conducted a focused inquiry into the primary postoperative discomfort symptom <pharyngalgia> pertaining to neck surgery. Notably, our investigation revealed no substantial differences across various dimensions, including the time lapse between infection and surgery or the levels of IgG antibodies. This outcome further solidifies our confidence in proceeding with thyroid surgery for well-prepared patients during the early phases of recovery. Furthermore, there exists a risk of systemic hypercoagulability and frequent venous thromboembolic events following a COVID infection. While certain reports indicate a prolongation of prothrombin time and other effects on coagulation function, severe bleeding events are infrequent.^[23–25]^ We have 1 patient who presented a decade-long history of headaches before surgery, with no preoperative abnormalities evident in brain imaging. However, this patient experienced an abrupt loss of consciousness 3 hours post-surgery. Subsequent CT scan examination confirmed cerebral hemorrhage, necessitating an emergency neurosurgical procedure. Although postoperative cerebral hemorrhage is exceedingly rare, its occurrence in our post-COVID infection patient underscores the importance of heightened vigilance, especially for patients with a pertinent medical history.

Limitations to the present study include that this is a single-center study, as the sample size may not be sufficiently large. Additionally, the majority of patients included in this study were at clinical stage I of the tumor, potentially affecting the generalizability of the research findings. It is worth considering whether the research conclusions would alter with the inclusion of patients at advanced stages. In conclusion, we recommend that the preoperative assessment of patients could incorporate IgG testing to be an indicator for assessing adverse surgical events, besides mere symptoms, lung CT scans, and COVID-polymerase chain reaction testing. With a thorough preoperative assessment, early thyroid surgery (even initial 2–4 week) following COVID-19 infection is feasible.

## Author contributions

**Data curation**: Mengjia Fei, Yuan Shi.

**Funding acquisition**: Mengjia Fei.

**Investigation**: Yuan Shi, Kai Guo.

**Methodology**: Kai Qian.

**Resources**: Yuan Shi, Kai Guo.

**Software**: Kai Qian.

**Supervision**: Zhuoying Wang.

**Validation**: Kai Guo, Zhuoying Wang.

**Visualization**: Zhuoying Wang.

**Writing – original draft**: Mengjia Fei.

**Writing – review & editing**: Zhuoying Wang.

## Supplementary Material
