# Supplementary material for: The influence of recent COVID-19 infection on patients undergoing thyroid surgery: Clinical outcomes and patient perception observations
Source: Medicine (Baltimore). 2026 May 12;104(49):e46400. doi: 10.1097/MD.0000000000046400 (PMC12688884; doi:10.1097/MD.0000000000046400)
Supplement: Supplementary file 1 [file medi-104-e46400-s001.pdf]

Survey Questionnaire: Post-Thyroid Surgery Throat Pain

Dear participant, thank you for taking part in our survey. This questionnaire aims to assess the severity and impact of throat pain after thyroid surgery. Please answer the following questions based on your actual condition.

Personal Information :

1. Gender:
   - Male
   - Female
   - Other

2. Age:
   - Under 18
   - 18-30
   - 31-50
   - 51-65
   - 65 and above

Pain Severity Assessment:

Please choose the phrase that best describes the severity of your throat pain:

3. **Pain Severity:**
   - 0 - No pain
   - 1 - Mild
   - 2 - Moderate
   - 3 - Moderately severe
   - 4 - Severe
   - 5 - Very severe

Pain Impact Assessment:

Please select the option that best represents your situation for the following questions:

4. Does throat pain bother you?
   - Yes
   - No

5. Impact of throat pain on daily activities:
   - No impact at all
   - Slight impact
   - Moderate impact

- Severe impact
- Very severe impact

6. Does throat pain affect your sleep?
   - Yes
   - No

7. Impact of throat pain on your emotional state:
   - No impact
   - Slight impact
   - Moderate impact
   - Severe impact
   - Very severe impact

**** Difference in throat pain between COVID-19 infection and post-surgery ****

8. How would you compare the throat pain you experienced after this recent thyroid surgery with the time when you had a COVID-19 infection?

- A little more painful this time
- Significantly more painful this time
- About the same for both occasions
- Throat pain was slightly painful during COVID-19 infection
- Throat pain was significantly more painful during COVID-19 infection

****Additional Information:****

9. Please describe the characteristics of your throat pain, such as the type of pain, duration, exacerbating or alleviating factors, etc.

10. Have you taken any measures to alleviate throat pain? Please briefly explain.

Thank you for your participation!

Thyroid Surgery Patient Symptom Survey

Dear respondent, we are collecting information regarding symptoms in patients after thyroid surgery to better understand post-operative conditions. Please answer the following questions honestly.

Personal Information:

1. Name (optional):
2. Age:
   - Under 18

- 18-30
- 31-50
- 51-65
- 65 and above

3. Your gender:
    - Male
    - Female
    - Other

Symptom Survey:

Please select the appropriate description for the following symptoms:

4. **Sore Throat:**
    - None
    - Mild
    - Moderate
    - Severe
5. **Cough:**
    - None
    - Occasionally
    - Frequently
    - Persistent
6. **Difficulty in Breathing:**
    - None
    - Mild
    - Moderate
    - Severe
7. **Duration of Symptoms:**
    - Few days
    - Within a week
    - One to two weeks
    - More than two weeks
8. **Weakness:**
    - None
    - Mild
    - Moderate
    - Severe

Additional Information:

9. Do you experience any other discomfort or symptoms after the surgery? Please briefly describe.
10. Did you seek medical help after the surgery? Please describe the actions you took.

Application and Approval Form for Ethical Review of Scientific Research

Project Application

一、Applicant fills in the section

**Basic research information**				
Declaration number	RA-2022-198			
entry name	A study on the time limit for thyroid tumor surgery after CIOVID-19 infection			
Project Source	Self drafted topic - clinical research			
Declaration date	September 10, 2022			
Main Researchers	Fei Mengjia	Responsible department	Head and neck surgery	
Do the main researchers have a GCP certificate and clinical trial experience			☒ Yes ◻ No	
Research nature	Multi center (☐ Team leader unit ☐ Participating units/Team leader:) ◦ Single center			
Research cycle	2 years (September 2022 to August 2024)			
Name of Team Researcher	Unit Name	Professional background	Professional title	Responsible matters
Fei Mengjia	Shanghai Jiao Tong University	Otolaryngology Head and Neck Surgery	Attending physician	Project design, research design, and paper writing
**Research specific content**				
research objective	The aim of this study is to explore the changes in relevant clinical, medical, and laboratory indicators before and after thyroid surgery at different time periods (2-8 weeks) after CIOVID-19 infection. We plan to search for the earliest suitable surgical opportunity.			
research method	☐ Intervention ◦ Observational (☐ Prospective ☒ Retrospective ☐ Current) Sample collection/Basic research			
Research object	☐ Healthy person ☒	sample size	two hundred	

		Patient (disease: papillary thyroid carcinoma)		
sample collection	type	☒ Blood Tissue other:	Whether to leave the country	Yes ☒No
	source	☒ Biological sample library<br>☐ Previous retention<br>Planned collection	international co-operation	☐Yes No
Name of medication/device/formulation used			Is it listed in China	Yes No
Does it exceed the dosage or method specified in the instructions for medication/device/formulation			Yes No No Not involved	
Does the medication/device/formulation exceed the indications specified in the instruction manual			Yes No No Not involved	
			◻ Yes ◻ No	
Whether to use placebo			Yes No No Not involved	
Is there any basic treatment available			Yes No No Not involved	
Reasons for the lack of basic treatment and the necessity of using placebo:				
Does it involve the following content:				
Vulnerable groups<br>Not involved		☐ Minors ☐ Pregnant women or fetuses ☒ Late stage tumor/cancer patients Mental disorders patients<br>☐ Lack of reading ability (illiteracy, visual impairment, intellectual disability, consciousness impairment, etc.) ☐ Elderly people<br>Other:		
		Reason for inclusion: necessity of tumor research		
Immunotherapy<br>No involvement		☐ Stem cells (Autologous Allogeneic) CAR-T cells PD-1/PD-L1 Other:		
Genetic content		◻ Not involved ☐ Involved (specific content:)		
High risk<br>New medical technologies<br>No involvement		Using large-scale instruments and equipment such as particle generators to implement destructive treatment technology<br>⑧ Radioactive particle implantation therapy technology ⑧ Tumor hyperthermia therapy technology ⑧ Tumor		

地址：上海市浦建路 160 号（200127）． 电话：（021）58752345－33364. 传真：（021）58394262

	cryotherapy technology<br>Artificial Intelligence Assisted Diagnosis and Treatment Technology Tissue and Cell Transplantation Technology Artificial Heart Implantation Technology<br>⑧ Tumor vaccine treatment technology ⑧ Homologous organ transplantation technology ⑧ Transgender surgery<br>⑧ Central nervous system surgery for drug rehabilitation<br>⑧ Gene therapy technology ⑧ Cloning therapy technology<br>⑧ Stereotactic surgery for the treatment of mental illness<br>⑧ Allogeneic stem cell transplantation technology<br>⑧ Autologous stem cell and immune cell therapy technology<br>⑧ Gene chip diagnosis and treatment technology<br>⑧ Surgical treatment techniques for bone fracture and height increase ⑧ Xenotransplantation techniques, etc

Declaration of Authenticity: The above content is personally filled out by me and is the actual content of this project application. If I intentionally conceal the research content and fail to fill it out according to the actual situation, resulting in a failure to pass the ethical review after project approval, I will bear the consequences myself.

Signature of main researcher		date	September 10, 2022

2、 Review decisions and opinions

Review comments
During the review process, it was not found that the research information and content provided by the researcher violated the social value of bioethics. The experimental plan was scientifically sound, and the proportion of trial risks and benefits to participants was reasonable. The researcher was able to fairly select participants, respect their rights, and make them fully aware. At the same time, the research team is set up reasonably, and the researchers and their team have sufficient research experience, qualifications, and time to complete the experiment. There is no record of bad research integrity standards, and they can safely and normatively carry out the study and ensure the safety and rights of the subjects. Moreover, the facilities and equipment of our institution meet the requirements of the experiment. Meets the basic requirements of ethical review.
Review decision

地址：上海市浦建路 160 号（200127）． 电话：（021）58752345－33364. 传真：（021）58394262

□□Agree to declare, disagree to declare	
Chairman	(Signature)
Ethics Committee	(Seal)
date	

地址：上海市浦建路 160 号（200127）. 电话：（021）58752345－33364. 传真：（021）58394262

上海交通大学医学院附属仁济医院医学伦理委员会

Shanghai Jiaotong University School of Medicine, Renji Hospital Ethics Committee

科研课题申报伦理审查申请及审批表

一、申请人填写部分

研究基本信息				
申报编号	RA-2022-198			
项目名称	有关 CIOVID-19 感染后进行甲状腺肿瘤手术的时限研究			
项目来源	自拟课题-临床研究			
申报日期	2022-09-10			
主要研究者	费梦嘉	承担科室	头颈外科	
主要研究者是否有 GCP 证书及临床试验经历			☒是 ☐否	
研究性质	☐多中心（☐组长单位 ☐参与单位/组长： ） ☒单中心			
研究周期	2 年（2022.09-2024.08）			
团队研究者姓名	单位名称	专业背景	职称	负责事项
费梦嘉	上海交通大学	耳鼻咽喉头颈外科学	主治医师	课题设计、研究设计、论文撰写

研究具体内容				
研究目的	本研究旨在探讨 CIOVID-19 感染后在不同时间段（2-8 周），进行甲状腺外科手术前后的相关临床医技实验室指标变化。拟寻找合适的最早的可手术时机。			
研究方法	☐干预 ☒观察性（☐前瞻性 ☒回顾性 ☐现况性） ☒样本采集/基础研究			
研究对象	☐健康人 ☒患者（疾病：甲状腺乳头状癌 ）		样本量	200
样本采集	类型	☒血液 ☒组织 ☐其他：	是否出境	☐是 ☒否
	来源	☐生物样本库☒既往留存 ☐计划采集	国际合作	☐是 ☒否
使用的药物/器械/制剂名称			是否在国内上市	☐是 ☐否
是否超出说明书规定剂量或方法用药/器械/制剂			☐是 ☐否 ☒不涉及	
是否超出说明书规定适应症用药/器械/制剂			☐是 ☐否 ☒不涉及	
是否有前期有效的临床研究/基础研究/动物实验支持			☒是 ☐否	
是否使用安慰剂			☐是 ☐否 ☒不涉及	
是否有基础治疗			☐是 ☐否 ☒不涉及	
✧ 无基础治疗且必须使用安慰剂的原因：				
是否涉及以下内容：				
弱势群体<br>☐不涉及	☐未成年人 ☐孕妇或胎儿 ☒晚期肿瘤/癌症患者 ☐精神障碍患者<br>☐无阅读能力（文盲，视力障碍，智力障碍，意识障碍等） ☐高龄老人<br>☐其他：			
	✧ 必须纳入的原因：肿瘤研究的必要性			
免疫疗法<br>☒不涉及	☐干细胞（☐自体 ☐异体） ☐CAR-T 细胞 ☐PD-1/PD-L1 ☐其他：			
遗传学内容	☒不涉及 ☐涉及（具体内容：）			
高风险的	☐利用粒子发生装置等大型仪器设备实施毁损式治疗技术			

地址：上海市浦建路 160 号（200127） 电话：（021）58752345—33364 传真：（021）58394262

医疗新技术<br>☒不涉及	☐放射性粒子植入治疗技术<br>☐人工智能辅助诊断治疗技术<br>☐瘤苗治疗技术<br>☐中枢神经系统手术戒毒<br>☐立体定向手术治疗精神病技术<br>☐自体干细胞和免疫细胞治疗技术<br>☐断骨增高手术治疗技术	☐肿瘤热疗治疗技术<br>☐组织、细胞移植技术<br>☐同种器官移植技术<br>☐基因治疗技术<br>☐异基因干细胞移植技术<br>☐基因芯片诊断和治疗技术<br>☐异种器官移植技术等	☐肿瘤冷冻治疗技术<br>☐人工心脏植入技术<br>☐变性手术<br>☐克隆治疗技术
**真实性申明：**以上内容由本人亲自填写，为本次申报课题的实际内容，若因本人故意隐瞒研究内容，未按实际情况填写导致立项后伦理审查不通过，后果由本人自己承担。			
主要研究者签字	费慧嘉	日期	2022. 09. 10

二、审查决定及意见

**审查意见**
在审查过程中未发现研究者提供的研究信息及内容违背生命伦理的社会价值，其试验方案符合科学性，试验风险及受试者获益比例合理，研究者能够公平的选择受试者，尊重其权利并使其充分知情。同时，研究团队设置合理，研究者及其团队具有充足的研究经验、资质及时间完成试验，且无不良科研诚信规范记录，能够安全规范地开展该项研究并确保受试者的安全及权益，且本机构的设施设备满足该试验需求。符合伦理审查的基本要求。
**审查决定**
☒同意申报　　　　☐不同意申报

主任委员	（签字）
伦理委员会	上海交通大学医学院附属仁济医院伦理委员会（盖章）
日期	2022-9-21

地址：上海市浦建路160号（200127）　电话：（021）58752345—33364　传真：（021）58394262
